# Entropy Analysis on the Blood Flow through Anisotropically Tapered Arteries Filled with Magnetic Zinc-Oxide (ZnO) Nanoparticles

**DOI:** 10.3390/e22101070

**Published:** 2020-09-24

**Authors:** Lijun Zhang, Muhammad Mubashir Bhatti, Marin Marin, Khaled S. Mekheimer

**Affiliations:** 1 College of Mathematics and Systems Science, Shandong University of Science and Technology, Qingdao 266590, China; li-jun0608@163.com; 2 Department of Mathematics and Computer Science, Transilvania University of Brasov, 500093 Brasov, Romania; m.marin@unitbv.ro; 3 Mathematical Department, Faculty of Science, Al-Azhar University, Nasr City, Cairo 11884, Egypt; kh_mekheimer@azhar.edu.eg

**Keywords:** entropy analysis, zinc-oxide nanoparticles (ZnO-NPs), blood flow, tapered artery, peristaltic flow

## Abstract

The present analysis deals with the entropy analysis of the blood flow through an anisotropically tapered arteries under the suspension of magnetic Zinc-oxide (ZnO) nanoparticles (NPs). The Jeffrey fluid model is contemplated as blood that is electrically conducting and incompressible. The lubrication approach is used for the mathematical modeling. The second law of thermodynamics is used to examine the entropy generation. The exact solutions are obtained against velocity and temperature profile with the use of computational software. The results for Entropy, Velocity, Bejan number, temperature profile, and impedance profile are discussed by plotting the graphs. ZnO-NPs have promising applications in biomedical engineering due to its low toxicity, economically reliable, and excellent biocompatibility. ZnO-NPs also emerged in medicine i.e., antibacterial and anticancer activity, and also beneficial in antidiabetic treatment. The monitoring of the blood temperature in the case of the tapered artery has supreme importance in controlling the temperature of blood in the living environment. The presence of a magnetic field is advantageous to manage and control the blood motion at different temperatures. The present outcomes are enriched to give valuable information for the research scientists in the field biomedical science, who are looking to examine the blood flow with stenosis conditions and also beneficial in treating multiple diseases.

## 1. Introduction

In recent years, nanomaterials in biomedical science have acquired significant attention because of their promising applications. The development of nanoparticles in a smaller size shows remarkable features in the biomedical field for anticancer gene/drug delivery, anti-bacteria, bio-sensing, and cell imaging. The usage of nanoparticles (NPs) is beneficial in a broad range i.e., diagnosis, imaging, and delivery. The unique features of magnetic nanoparticles (MNPs) are useful in theranostics (magnetic resonance imaging agents), magnetic drug targeting, and magnetic fluid hyperthermia vehicles. There are two components of nanoparticles, i.e., the surface modifier and the core material. The core material is made up of biological materials i.e., lipids, phospholipids, chitosan, dextran, and lactic acid, or maybe made up of metals, carbon, silica, and chemical polymers. The surface modifier is accountable for the change in the physiochemical features of core materials. Multiple amounts of chemical compounds, probs, drugs, and proteins are connected to the nanoparticle surface by adsorption process or due to covalent bonds’ assistance.

Zinc oxide (ZnO) nanoparticles (NPs) is one of the essential nanoparticles that are applicable in multiple fields because of their promising chemical and physical features [1,2]. ZnO NPs were firstly used in the rubber industry [3,4], but later it was also used in various cosmetic products [5]. Apart from the applications mentioned earlier, ZnO NPs are applicable in multiple industrial processes i.e., photocatalysis, electronics, electro-technology industries, and concrete production [3,6]. ZnO NPs consist of low-toxicity and low-cost nanomaterial, which is beneficial in antioxidant, antibacterial, anticancer, anti-inflammatory, and antidiabetic activities as well as useful in bio-imaging and drug delivery [7,8,9].

Sucharitha et al. [10] discussed the peristaltic nanofluid flow with magnetic and Joule heating effects. Shahzadi et al. [11] contemplated the impact of carbon nanotubes propagating in a wavy annulus with variable viscosity features. Mekheimer et al. [12] used a third-grade fluid model to determine the behavior of gold nanoparticles suspended in blood and presented an application associated with cancer therapy. Eldabe et al. [13] also considered the gold nanoparticles in blood flow but contemplated the non-Darcy porous medium. Prakash et al. [14] presented an application of blood flow using the peristaltic pumping model through a tapered channel filled with nanofluids. Ebaid et al. [15] used a homotopy perturbation scheme to determine the behavior of gold nanoparticles suspended in blood and moving in sinusoidal format. Khan et al. [16] investigated multiple shapes of nanoparticles through an asymmetric peristaltically induced channel under magnetic effects. Ali et al. [17] investigated the hybrid TiO${}_{2}$ and Cu-H${}_{2}$O nanofluid under slip and magnetic forces via the peristaltic mechanism. Some recent studies associated with the current topic can be found from the references [18,19,20,21,22].

Entropy is one of the essential topic and plays a crucial role in our daily life. It is associated with the second law of thermodynamics [23], which provides a measure of system’s disorder. According to the thermodynamics, the physical process can be classified into two parts: irreversible and reversible. A zero change in the entropy reveals the reversible process, whereas, when it is different from zero, it reveals the irreversible process. Thus, the entropy generation can be contemplated as the measure of the irreversibility of a process.

Different researchers examined the entropy generation with heat transfer. For instance, Akbar et al. [24] discussed the peristaltic propulsion of copper water (H${}_{2}$O+Cu) nanofluid flow with thermal conductivity and presented a detailed analysis of entropy generation. Ellahi et al. [25] studied the peristaltic motion of nanofluid through a porous medium using Darcy law. Ranjit and Shit [26] contemplated the electro-osmotic flow with entropy generation and discussed the peristaltic pumping under magnetic effects. Qasim et al. [27] presented a detailed analysis of entropy generation on a wavy channel filled with methanol-based nanofluid. Shehzad et al. [28] presented a mathematical model of the entropy generation using non-Darcy Poiseuille flow with the application of purification. Jangili and Bég [29] investigated the entropy generation in a micropolar fluid propagating through a vertical plate under magnetic and buoyancy effects. Ali et al. [30] contemplated the similar problem [24] but with slip effects. Saleem and Munawar [31] studied the cilia motion and entropy generation with a non-Newtonian fluid model using Ohm’s law. Noreen et al. [32] studied the entropy generation on the peristaltically induced motion with hall current and ohmic heating. Narla et al. [33] explored entropy generation in electro-osmotic nanofluid flow in a curvy channel with joule dissipation. Monaledi and Makinde [34] investigated the entropy generation in a microchannel filled with nanoparticles and propagating via Poiseuille flow mechanism. Riaz et al. [35] presented a detailed mathematical analysis of the peristaltic asymmetric wavy motion of blood with entropy generation with convection.

Due to such promising applications of ZnO NPs, the present study’s main target is to discuss the entropy generation on the blood flow through anisotropically tapered arteries filled with magnetic Zinc-oxide (ZnO) nanoparticles.The ZnO nanoparticles play a significant role in anticancer effects. Magnetic NPs are beneificial for synergic actions as well as direct heating, and the killing of the cancer cells. Furthermore, Magnetic NPs are important in magnetic drug targeting, targeted delivery, and magnetic hyperthermia. A significant motivation to examine the flow through the converging-diverging artery comes from medical science. In the mammals’ arterial systems, it is usual to observe stenosis or narrowings, including axisymmetric or collar-like. These constrictions are because of the impingement of ligaments, intravascular plaques, and spurs on the wall’s vessel [36]. Once the vascular lesion has been evolved, a coupling impact occurs betwixt its further production and the change of flow features [37]. The understanding of the flow in the neighborhood of the stenosis is beneficial to examine the important complications that occur due to such contractions. For instance, the inner generation of tissue into the artery, the production of thrombus, and the bulging and the weakening of the artery downward from the stenosis.

For the proposed flow, the Jeffrey fluid model with incompressible and electrically conducting features have been contemplated. The Jeffrey fluid model gives dual behavior, i.e., Newtonian and non-Newtonian. The proposed Jeffrey fluid model is adequate to express the stress relaxation features of non-Newtonian fluids, which usual viscous fluid models fails to express. The lubrication theory and the second law of thermodynamics are applied to formulate mathematical modeling. The extrinsic magnetic field is also contemplated; in addition, the behavior of viscous dissipation and Joule heating are also contemplated with an energy equation. The exact solutions are obtained against velocity and temperature profile with the use of computational software Mathematica using a Built-in command ‘DSolve’. The significant results are discussed across all the leading parameters.

## 2. Problem Description and Modeling

**Figure 1:**
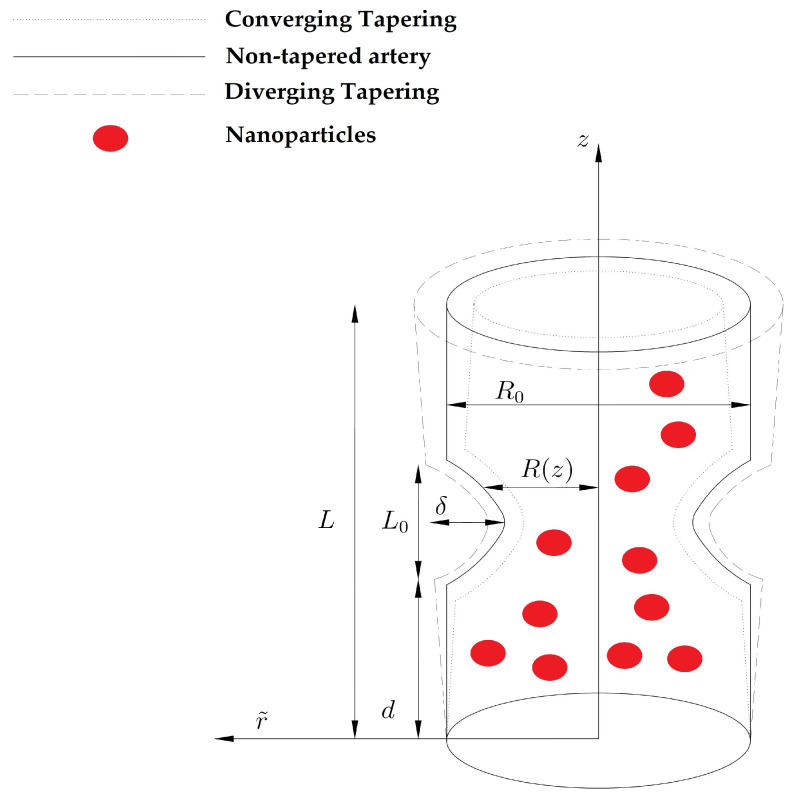
Geometrical configuration of the anisotropically tapered artery.

Let us contemplate a finite tube having length *L* filled with non-Newtonian fluid and Zinc-oxide (ZnO) NPs. An extrinsic magnetic field is applied while the induced magnetic field is assumed to be negligible to small magnetic Reynolds number. The non-Newtonian contains the following features i.e., irrotational, constant density, electrically conducting, and incompressible. We have contemplated the cylindrical polar coordinates $\tilde{r},\tilde{\theta },\tilde{z}$ while $\tilde{r}$ lies towards the radial direction, $\tilde{\theta }$ is located along the circumferential direction, and $\tilde{z}$ is contemplated along the axis of the artery as displayed in Figure 1. Furthermore, $\tilde{r}=0$ represents the axis of the tube. The presence of heat transfer is also contemplated. At the wall of the tube, the temperature ${\tilde{T}}_{1}$ is assumed. The mathematical expression for the proposed anisotropically tapered artery with time-variant features is described as:

(1) $$\begin{array}{c} R\left(\tilde{z}\right)=\left\{\begin{array}{c} \ell \left(\tilde{t}\right)\left[\tilde{z}\eta +R_{0}-\frac{\delta \cos\psi }{L_{0}}\left(11-\frac{94}{3L_{0}}\hbar +\frac{32}{L_{0}^{2}}\hbar^{2}-\frac{32}{L_{0}^{3}}\hbar^{3}\right)\right];d\leq \tilde{z}\leq d+\frac{3L_{0}}{2}\\ \ell \left(\tilde{t}\right)\left(1+\tilde{z}\eta \right);\;\;\;\;\;\;\;\;\;\;\;\;\;\;\;\;\;\;\;\;\;\;\;\;\;\;\;\;\;\;\;\;\;\;\;\;\;\;\;\;\;\;\;\;\;\;\;\mathrm{otherwise} \end{array}\right., \end{array}$$

where $\hbar =\tilde{z}-d$, $R_{0}$ represents the radius of the normal artery contains the non-stenotic area, the stenotic length is denoted by $L_{0}$, the height of the stenosis is δ, $\tilde{t}$ the time, $R(\tilde{z})$ represents the artery radius and the tapered arterial segment having composite stenosis, and the tapering angle is denoted by ψ, and η=tanψ denotes the slope of tapered vessel.

We determine three distinct shapes of artery i.e., the diverging tapered artery (ψ>0), the converging tapered artery (ψ<0), and the non-tapered artery (ψ=0). It can be expressed in mathematical form as:

(2) $$\begin{array}{c} \psi =\left\{\begin{array}{c} \mathrm{Converging}\;\mathrm{artery}\;\;\;\;\;\;\;\psi <0,\\ \text{Non-tapered artery}\;\;\;\;\psi =0,\\ \mathrm{Diverging}\;\mathrm{artery}\;\;\;\;\;\;\;\;\;\psi >0, \end{array}\right. \end{array}$$

The internal growth of the tissues, thrombus, in the artery is responsible for the diverging case. The inner development of the tissues in the artery provides significant resistance and provides resistance to the flow. The tissue may grow until the artery gets wholly occluded. The emergence of mural thrombi at the position of the narrowed artery causes the same similar obstacles as the internal growth of the tissues. However, a non-tapered case reveals that the artery is smooth and uniform throughout the whole region. The converging case occurs due to atherosclerotic plaques, which usually occurs due to faulty lipid metabolism. Plaques consist of lipids and are often found these days in the arteries, which occurs due to high cholesterol diet, etc.

The time-variant function $\ell (\tilde{t})$ reads as

(3) $$\begin{array}{c} \ell \left(\tilde{t}\right)=\frac{e^{\omega \alpha \tilde{t}}+\left(1-\cos\omega \tilde{t}\right)\alpha }{e^{\omega \alpha \tilde{t}}}, \end{array}$$

where ω represents the radial frequency due to force oscillation, and α the constant.

The proposed Jeffrey fluid model is

(4) $$\begin{array}{cc} \; & \tau =\frac{\mu_{nf}}{1+\Gamma_{1}}\left(\dot{\vartheta }+\Gamma_{2}\ddot{\vartheta }\right), \end{array}$$

and

(5) $$\begin{array}{c} \mu_{nf}=\frac{\mu_{f}}{{\left(1-\Psi \right)}^{2.5}}, \end{array}$$

where the nanofluid viscosity is $\mu_{nf}$, Ψ indicates the nanoparticle volume fraction, $\Gamma_{1}$ the ratio betwixt the relaxation to retardation time, $\Gamma_{2}$ denotes the delay time, ϑ the shear rate, and dots represent the differentiation w.r.t time.

In component form, they are found as

(6) $$\begin{array}{c} \tau_{\tilde{r}\tilde{r}}=\frac{2\mu_{nf}}{1+\Gamma_{1}}\left[1+\Gamma_{2}\left(v\frac{\partial }{\partial \tilde{r}}+\tilde{u}\frac{\partial }{\partial \tilde{z}}\right)\right]\frac{\partial \tilde{v}}{\partial \tilde{r}}, \end{array}$$

(7) $$\begin{array}{c} \tau_{\tilde{r}\tilde{z}}=\tau_{\tilde{z}\tilde{r}}=\frac{\mu_{nf}}{1+\Gamma_{1}}\left[1+\Gamma_{2}\left(v\frac{\partial }{\partial \tilde{r}}+\tilde{u}\frac{\partial }{\partial \tilde{z}}\right)\right]\left(\frac{\partial \tilde{v}}{\partial \tilde{z}}+\frac{\partial \tilde{u}}{\partial \tilde{r}}\right), \end{array}$$

(8) $$\begin{array}{c} \tau_{\tilde{r}\tilde{r}}=\frac{2\mu_{nf}}{1+\Gamma_{1}}\left[1+\Gamma_{2}\left(v\frac{\partial }{\partial \tilde{r}}+\tilde{u}\frac{\partial }{\partial \tilde{z}}\right)\right]\frac{\partial \tilde{u}}{\partial \tilde{z}}. \end{array}$$

The continuity equation, equation of motion with body forces, and energy equation are described as [38]

(9) $$\begin{array}{c} \frac{1}{\tilde{r}}\frac{\partial }{\partial \tilde{r}}\left(\tilde{r}\tilde{v}\right)+\frac{\partial \tilde{u}}{\partial z}=0, \end{array}$$

(10) $$\begin{array}{c} \rho_{nf}\left(\tilde{v}\frac{\partial \tilde{v}}{\partial \tilde{r}}+\tilde{u}\frac{\partial \tilde{v}}{\partial \tilde{z}}\right)=-\frac{\partial \tilde{p}}{\partial \tilde{r}}+\frac{1}{\tilde{r}}\frac{\partial }{\partial \tilde{r}}\tilde{r}\tau_{\tilde{r}\tilde{r}}+\frac{\partial }{\partial \tilde{r}}\tau_{\tilde{r}\tilde{z}}-\frac{1}{\tilde{r}}\tau_{\tilde{\theta }\tilde{\theta }}, \end{array}$$

(11) $$\begin{array}{c} \rho_{nf}\left(\tilde{v}\frac{\partial \tilde{u}}{\partial \tilde{r}}+\tilde{u}\frac{\partial \tilde{u}}{\partial \tilde{z}}\right)=-\frac{\partial \tilde{p}}{\partial \tilde{z}}+\frac{1}{\tilde{r}}\frac{\partial }{\partial \tilde{r}}\tilde{r}\tau_{\tilde{r}\tilde{r}}+\frac{\partial }{\partial \tilde{r}}\tau_{\tilde{z}\tilde{z}}-\sigma_{nf}B_{0}^{2}\tilde{u}, \end{array}$$

(12) $$\begin{array}{c} {\left(\rho c_{p}\right)}_{nf}\left(\tilde{v}\frac{\partial \tilde{T}}{\partial \tilde{r}}+\tilde{u}\frac{\partial \tilde{T}}{\partial \tilde{z}}\right)=\kappa_{nf}\left(\frac{\partial^{2}\tilde{T}}{\partial {\tilde{r}}^{2}}+\frac{1}{\tilde{r}}\frac{\partial \tilde{T}}{\partial \tilde{r}}+\frac{\partial^{2}\tilde{T}}{\partial {\tilde{z}}^{2}}\right)+\tau_{\tilde{r}\tilde{z}}\frac{\partial \tilde{u}}{\partial \tilde{r}}+\sigma_{nf}B_{0}^{2}{\tilde{u}}^{2}, \end{array}$$

and [39]

(13) $$\begin{array}{c} \frac{\sigma_{nf}}{\sigma_{f}}=1+\frac{3\Psi \left(\bar{\sigma }-1\right)}{\left(\bar{\sigma }+2\right)-\Psi \left(\bar{\sigma }-1\right)},\bar{\sigma }=\frac{\sigma_{np}}{\sigma_{f}},\frac{\kappa_{nf}}{\kappa_{f}}=\frac{\kappa_{np}+2\kappa_{f}-2\Psi \left(\kappa_{f}-\kappa_{np}\right)}{\kappa_{np}+2\kappa_{f}+2\Psi \left(\kappa_{f}-\kappa_{np}\right)}, \end{array}$$

where ${\left(\rho c_{p}\right)}_{nf}$ the specific heat capacity of nanofluid, $\rho_{nf}$ the density of nanofluid, $\sigma_{nf}$ the electrical conductivity of nanofluid, $\sigma_{np}$ the electrical conductivity of the NPs $\kappa_{nf}$ the thermal conductivity of nanofluid, $\kappa_{p}$ the thermal conductivity of NPs, and $B_{0}$ the applied magentic field. The values used in Equation (13) are presented in Table 1. The boundary conditions according to the proposed flow configuration are described as

(14) $$\begin{array}{cc} \; & \frac{\partial \tilde{u}}{\partial \tilde{r}}=\frac{\partial \tilde{T}}{\partial \tilde{r}}=0,\;\;\;\;\tilde{r}=0, \end{array}$$

(15) $$\begin{array}{cc} \; & \tilde{u}=\tilde{0},\tilde{T}={\tilde{T}}_{1},\;\;\;\;\tilde{r}=R\left(\tilde{z}\right). \end{array}$$

The following are the non-dimensional quantities which are helpful for further formulation

(16) $$\begin{array}{cc} \; & r=\frac{\tilde{r}}{R_{0}},u=\frac{\tilde{u}}{U},z=\frac{\tilde{z}}{L_{0}},v=\frac{L_{0}}{\delta U}\tilde{v},L=\frac{\tilde{L}}{L_{0}},R=\frac{R}{R_{0}},p=\frac{R_{0}^{2}}{\mu_{f}L_{0}U}\tilde{p},\eta =\frac{\eta L_{0}}{R_{0}},\\ \; & \tilde{T}={\tilde{T}}_{1}+T\left({\tilde{T}}_{0}-{\tilde{T}}_{1}\right),\delta =\frac{\delta }{R_{0}}. \end{array}$$

Using the above equations in the governing equations, and an appropriate use of lubrication theory, the mathematical modeling leads to the following formulation:

(17) $$\begin{array}{c} \frac{\partial p}{\partial r}=0, \end{array}$$

(18) $$\begin{array}{c} \frac{dp}{dz}=\frac{\mu_{nf}}{r\mu_{f}\left(\Gamma_{1}+1\right)}\frac{\partial }{\partial r}\left(r\frac{\partial u}{\partial r}\right)-\frac{\sigma_{nf}}{\sigma_{f}}Ha^{2}u. \end{array}$$

In the above equation, the results for Newtonian fluid model reduce for $\Gamma_{1}=0.$

(19) $$\begin{array}{c} \frac{\kappa_{nf}}{\kappa_{f}}\frac{\partial^{2}T}{\partial r^{2}}+\frac{\mu_{nf}B_{m}}{\mu_{f}\left(\Gamma_{1}+1\right)}{\left(\frac{\partial u}{\partial r}\right)}^{2}+\frac{\sigma_{nf}}{\sigma_{f}}B_{m}Ha^{2}u^{2}=0, \end{array}$$

where Ha is the Hartmann number and $B_{m}$ the Brinkman number which is found as

(20) $$\begin{array}{c} Ha=\sqrt{\frac{\sigma_{f}}{\mu_{f}}}B_{0}R_{0},B_{m}=\frac{\mu_{f}U^{2}}{\kappa_{f}\left({\tilde{T}}_{0}-{\tilde{T}}_{1}\right)}. \end{array}$$

The boundary conditions become

(21) $$\begin{array}{cc} \; & \frac{\partial u}{\partial r}=\frac{\partial T}{\partial r}=0,\;\;\;\;r=0, \end{array}$$

(22) $$\begin{array}{cc} \; & u=0,T=0,\;\;\;\;r=R(z). \end{array}$$

## 3. Entropy Generation Analysis

The volumetric entropy generation in dimensional form reads as [40,41,42]

(23) $$\begin{array}{c} E_{\mathrm{gen}}^{{}^{\prime \prime \prime }}=\frac{\kappa_{n}f}{{\tilde{T}}_{0}}{\left(\frac{\partial \tilde{T}}{\partial \tilde{r}}\right)}^{2}+\frac{\tau_{\tilde{r}\tilde{z}}}{{\tilde{T}}_{0}}\left(\frac{\partial \tilde{u}}{\partial \tilde{r}}\right)+\frac{\sigma_{nf}B_{0}^{2}}{{\tilde{T}}_{0}}{\tilde{u}}^{2}, \end{array}$$

The above equations are comprised of three parts. The first term on the right-hand side represents irreversibility due to heat transfer, the second term shows the irreversibility process due to fluid friction, and the last term shows the behavior of hydromagnetics.

Applying the dimensionless variables in Equation (16) to the above equation, we obtain the following form of the entropy equation

(24) $$\begin{array}{c} E_{s}=\frac{E_{\mathrm{gen}}^{{}^{\prime \prime \prime }}}{E_{g}^{{}^{\prime \prime \prime }}}=\frac{\kappa_{nf}}{\kappa_{f}}{\left(\frac{\partial T}{\partial r}\right)}^{2}+\frac{{\bar{T}}_{0}B_{m}}{\Gamma_{1}+1}\left(\frac{\mu_{nf}}{\mu_{f}}\right){\left(\frac{\partial u}{\partial r}\right)}^{2}+\frac{\sigma_{nf}}{\sigma_{f}}B_{m}{\bar{T}}_{0}Ha^{2}u^{2}, \end{array}$$

where

(25) $$\begin{array}{c} E_{g}^{{}^{\prime \prime \prime }}=\frac{\kappa_{f}\left({\tilde{T}}_{0}-{\tilde{T}}_{1}\right)}{{\bar{T}}_{0}^{2}R_{0}^{2}},{\bar{T}}_{0}=\frac{{\tilde{T}}_{0}}{{\tilde{T}}_{0}-{\tilde{T}}_{1}}. \end{array}$$

Brinkman number $B_{m}$, and the Hartmann number Ha is defined in Equation (20). The Bejan number for the present formulation reads as

(26) $$\begin{array}{c} N_{b}=\frac{\frac{\kappa_{nf}}{\kappa_{f}}{\left(\frac{\partial T}{\partial r}\right)}^{2}}{\frac{\kappa_{nf}}{\kappa_{f}}{\left(\frac{\partial T}{\partial r}\right)}^{2}+\frac{{\bar{T}}_{0}B_{m}}{\Gamma_{1}+1}\left(\frac{\mu_{nf}}{\mu_{f}}\right){\left(\frac{\partial u}{\partial r}\right)}^{2}+\frac{\sigma_{nf}}{\sigma_{f}}B_{m}{\bar{T}}_{0}Ha^{2}u^{2}}. \end{array}$$

## 4. Solution of the Problem

The formulated Equations (18) and (19) are linear but coupled differential equations. Therefore, utilization of computational software *Mathematica 10.3*v is helpful to solve these kinds of differential equations. The results are obtained by utilizing the built-in command in *Mathematica*. We obtain the exact solutions as:

(27) $$\begin{array}{cc} \; & u=\frac{1}{A_{2}Ha^{2}}\frac{dp}{dz}\left(\frac{I_{0}\left(u_{0}r\right)-I_{0}\left(u_{0}R\right)}{I_{0}\left(u_{0}R\right)}\right), \end{array}$$

(28) $$\begin{array}{cc} \; & T=B_{m}{\left(\frac{dp}{dz}\right)}^{2}\left[-2\left\{A_{1}\left(1+\Gamma_{1}\right)+A_{2}Ha^{2}r^{2}\Gamma_{1}\right\}I_{0}{\left(u_{0}r\right)}^{2}-\left\{2A_{1}\left(1+\Gamma_{1}\right)\left(3+4\Gamma_{1}\right)\right.\right.\\ \; & \left.+A_{2}Ha^{2}\left(r^{2}\left(1+\Gamma \right)-R^{2}\left(1+3\Gamma_{1}\right)\right)\right\}I_{0}{\left(u_{0}R\right)}^{2}+2A_{2}Ha^{2}\Gamma_{1}\left(r^{2}I_{0}\left(u_{0}r\right)-R^{2}I_{0}\left(u_{0}R\right)\right)\\ \; & \times I_{0}\left(u_{0}r\right)\left\{8A_{1}{\left(1+\Gamma_{1}\right)}^{2}I_{0}\left(u_{0}R\right)+A_{2}Ha^{2}\Gamma_{1}r^{2}{}_{0}F_{1}\left(;2;\frac{u_{0}^{2}}{4}r^{2}\right)\right\}\\ \; & \left.-A_{2}Ha^{2}R^{2}\Gamma_{1}I_{0}\left(u_{0}R\right)\frac{1}{\Gamma (2)}{}_{0}F_{1}\left(2;\frac{u_{0}^{2}}{4}R^{2}\right)\right]÷4A_{2}^{2}A_{3}Ha^{4}\left(1+\Gamma_{1}\right)I_{0}{\left(u_{0}R\right)}^{2}, \end{array}$$

where

(29) $$\begin{array}{c} u_{0}=\frac{\sqrt{A_{2}}Ha}{\sqrt{A_{1}\left(1+\Gamma_{1}\right)}},A_{1}=\frac{\mu_{nf}}{\mu_{f}},A_{2}=\frac{\sigma_{nf}}{\sigma_{f}},A_{3}=\frac{\kappa_{nf}}{\kappa_{f}}. \end{array}$$

In the above equations, $I_{0}$ is the Bessel functions of zeroth-order, and ${}_{0}F_{1}$ represents the hypergeometric function.

The flux is calculated utilizing the following expression:

(30) $$\begin{array}{c} Q=2r\int_{0}^{R}udr, \end{array}$$

(31) $$\begin{array}{c} Q=\frac{dp}{dz}\frac{R^{2}}{A_{2}Ha^{2}I_{0}\left(u_{0}R\right)}\left[{}_{0}F_{1}\left(2;\frac{u_{0}^{2}}{4}R^{2}\right)-I_{0}\left(u_{0}R\right)\right]. \end{array}$$

The impedance is calculated utilizing the following expression

(32) $$\begin{array}{c} \Lambda =\frac{1}{Q}\int_{0}^{L}\left(-\frac{dp}{dz}\right)dr. \end{array}$$

where

(33) $$\begin{array}{c} \frac{dp}{dz}=\frac{A_{2}Ha^{2}QI_{0}\left(u_{0}R\right)}{R^{2}\left[I_{0}\left(u_{0}R\right)-{}_{0}F_{1}\left(2;\frac{u_{0}^{2}}{4}R^{2}\right)\right]}. \end{array}$$

## 5. Graphical Analysis

In this section, the graphical outcomes are elaborated against all the leading parameters for velocity, temperature, entropy, and Bejan number profile. The following are the parameter values that are used to elaborate the numerical results: $L_{0}=1;$ Ψ=0.1; $B_{m}=0.2;$ $\Gamma_{1}=1;$ ω=0.2; α=0.7; Ha=4; δ=0.2, and the thermophysical properties for blood and ZnO-NPs are given in Table 1. All the graphical results are plotted for three distinct cases i.e., converging ψ<0, non-tapered ψ=0, and diverging ψ>0.

**Table 1:** Thermo-physical properties of blood and Zinc-oxide NPs [43,44].

Physical Properties	$c_{p}$ (J/Kg·K)	ρ (Kg/m${}^{3}$)	κ (W/mK)
ZnO	523	5700	25
Blood	1063	3594	0.492

**Figure 2:**
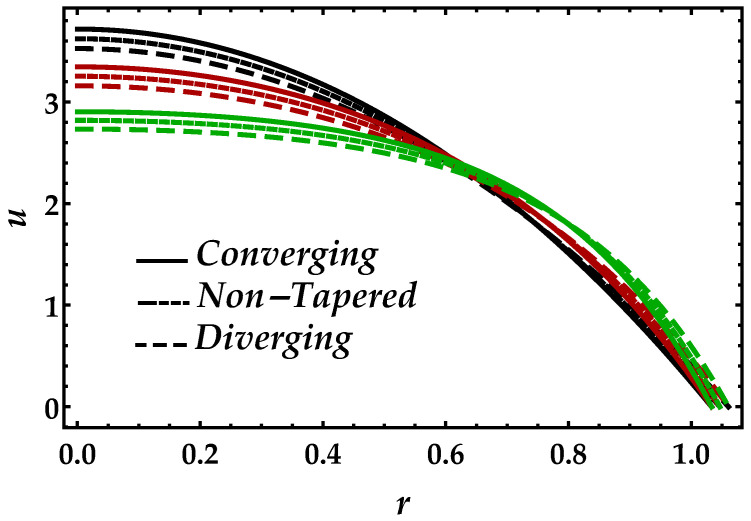
Consequences of velocity against multiple values of Ha. Black color: Ha=1; red color: Ha=4; green color: Ha=7.

**Figure 3:**
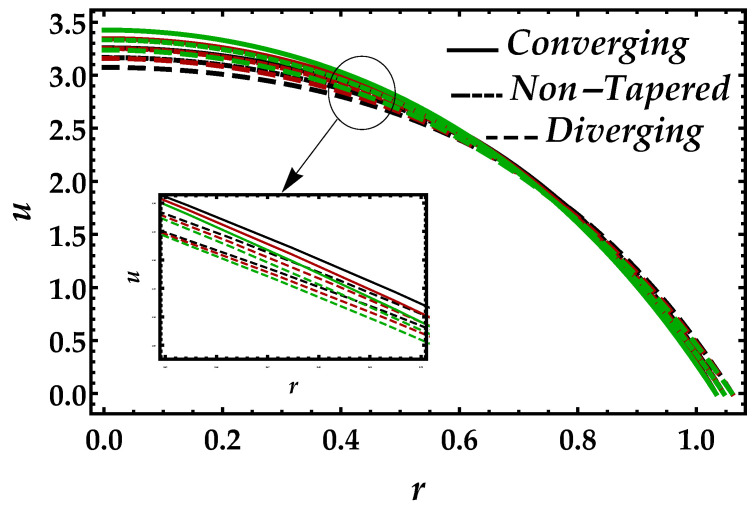
Consequences of velocity against multiple values of Ψ. Black color: Ψ=0; red color: Ψ=0.1; green color: Ψ=0.2.

**Figure 4:**
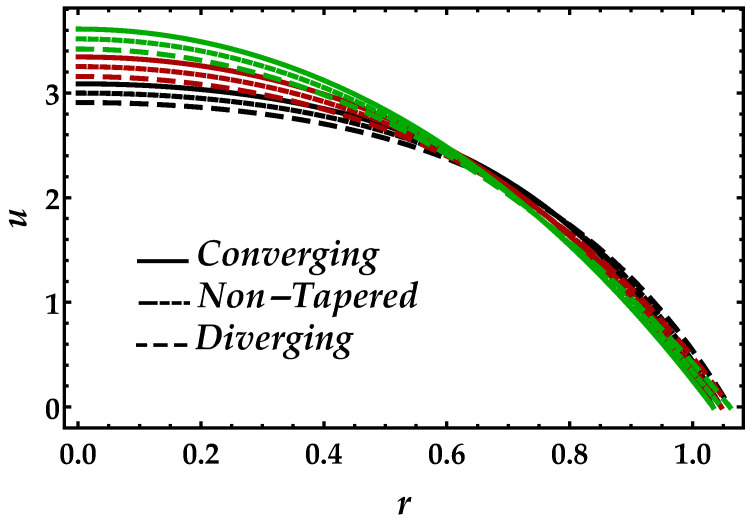
Consequences of velocity against multiple values of $\Gamma_{1}$. Black color: $\Gamma_{1}=0$; red color: $\Gamma_{1}=1$; green color: $\Gamma_{1}=6$.

**Figure 5:**
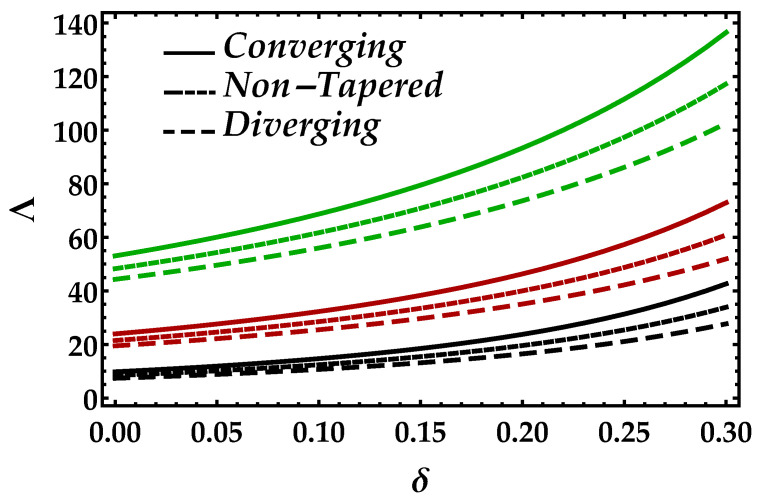
Consequences of impedance vs. δ against multiple values of Ha. Black color: Ha=1; red color: Ha=4; green color: Ha=7.

**Figure 6:**
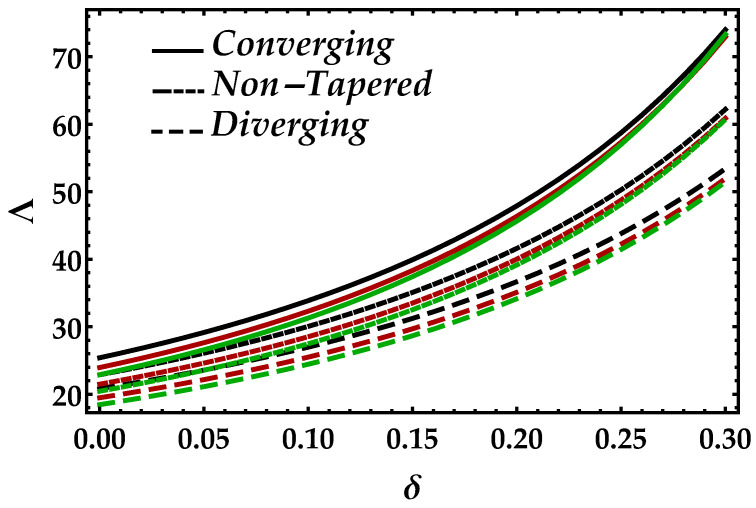
Consequences of impedance vs. δ against multiple values of Ψ. Black color: Ψ=0; red color: Ψ=0.1; green color: Ψ=0.2.

Figure 2 is sketched to observe the behavior of the magnetic field Ha on the motion of the blood under the suspension of the magnetic field. The response is dual in the artery; for instance, along the walls, it increases, whereas it decreases in the middle of the artery. This shows that the Lorentz force, which occurs due to the magnetic field, is more effective in the middle of the artery. The behavior of the blood flow in all three cases i.e., diverging, non-tapered, and converging, is the same as the effects of the magnetic field. Figure 3 shows the effects of nanoparticle volume Ψ on the velocity of blood for all the cases. It is easily observable that, in the middle of the artery, the blood flow gains its maximal velocity due to the increment in nanoparticle volume fraction; however, closer to the artery, it decreases. Furthermore, Ψ reflects the results for single-phase motion. Another thing we can see is that the effects of Ψ are small. Figure 4 presents the behavior of Jeffrey fluid parameter $\Gamma_{1}$ on the velocity profile against all three cases. In this figure, the results for Newtonian fluid $\Gamma_{1}=0$ are also plotted. It can be seen that Jeffrey fluid parameter enhances the velocity of the fluid in the middle of the channel, while the effects closer to the walls are negligible. Figure 5 shows the consequences of impedance profile Λ versus the height of stenosis δ against distinct values of Ha for all the cases of proposed geometry. In this figure, we can see that, by increasing magnetic effects, the impedance profile uniformly increases; however, less magnitude has been observed for diverging and non-tapered cases than the converging artery. Next, Figure 6 represents the variation of Ψ on the impedance profile. In this figure, we found that, for the single-phase case when Ψ=0, the magnitude of the impedance profile is maximal, whereas, by increasing the values of Ψ, the impedance profile reduces.

**Figure 7:**
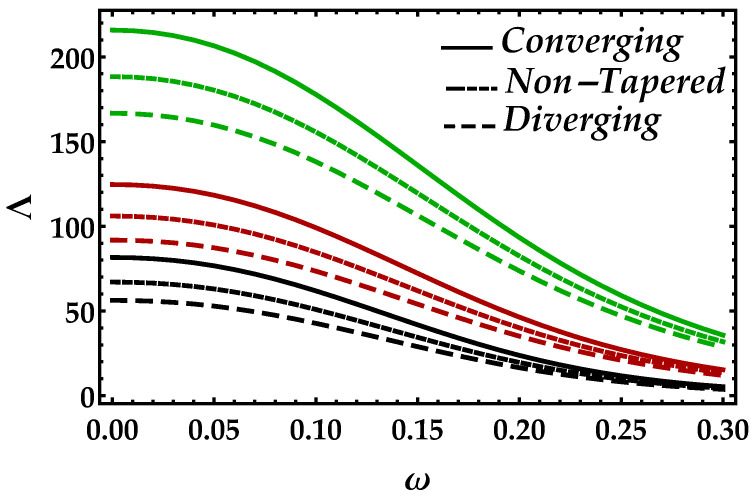
Consequences of impedance vs. ω against multiple values of Ha. Black color: Ha=1; red color: Ha=4; green color: Ha=7.

**Figure 8:**
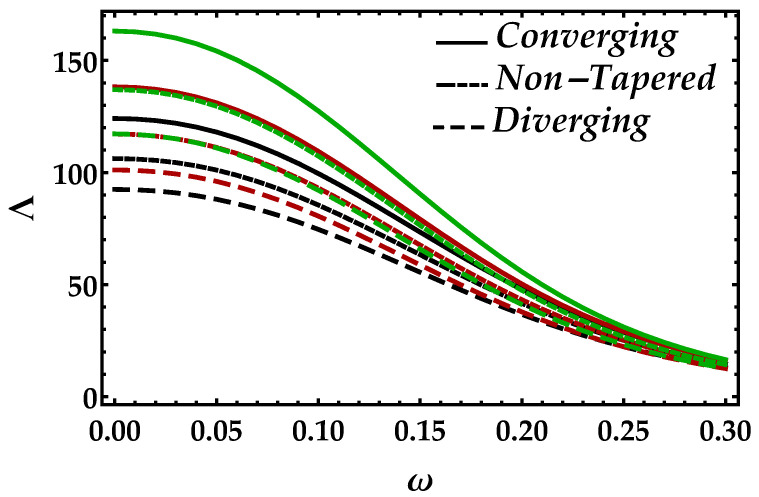
Consequences of impedance vs. ω against multiple values of Ψ. Black color: Ψ=0; red color: Ψ=0.1; green color: Ψ=0.2.

Figure 7 is plotted to determine the consequences of impedance Λ versus radial frequency ω for multiple values of Ha. In this figure, we can see in the horizontal directional that, as ω→0.3, the impedance profile is decreasing, which shows that higher values of radial frequency are less effective on the impedance of blood. Furthermore, the Lorentz force that occurs due to the magnetic field boosted the impedance profile throughout the domain; however, it is decreasing against diverging and non-tapered cases. It depicts from Figure 8 that an increment in Ψ significantly enhances the impedance profile; however, the results for all the cases and every value of Ψ are negligible when then the radial frequency approaches to 0.3.

**Figure 9:**
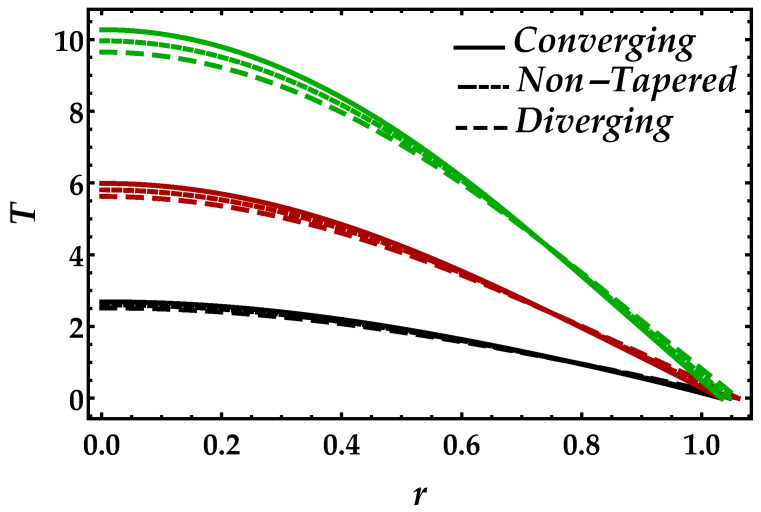
Consequences of temperature against multiple values of Ha. Black color: Ha=3; red color: Ha=5; green color: Ha=7.

**Figure 10:**
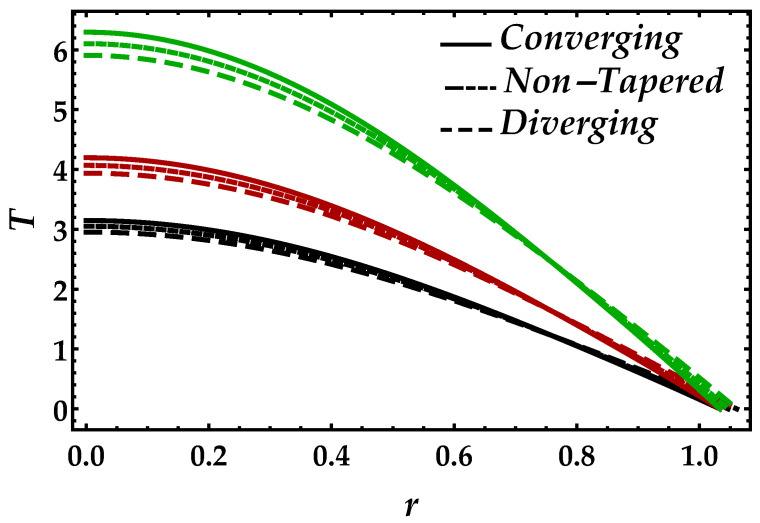
Consequences of temperature against multiple values of $B_{m}$. Black color: $B_{m}=0.1$; red color: $B_{m}=0.2$; green color: $B_{m}=0.3$.

**Figure 11:**
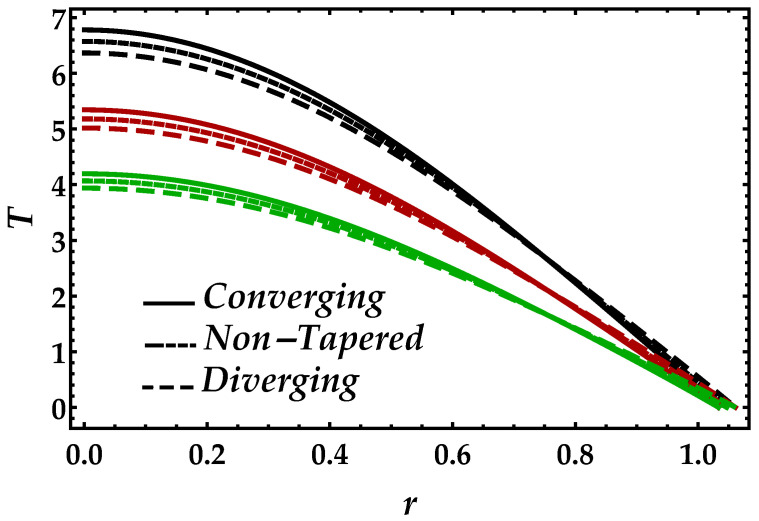
Consequences of temperature against multiple values of Ψ. Black color: Ψ=0; red color: Ψ=0.05; green color: Ψ=0.1.

Figure 9 demonstrates the behavior of the magnetic field on the temperature profile. From this figure, we can observe that the pattern gets higher in magnitude when the effects of the magnetic field increases. Another thing we can see is that, when the magnetic field is small i.e., Ha=3, the converging, diverging, and non-tapered artery shows similar behavior, but, by increasing the magnetic field, the results become more transparent. Figure 10 is plotted for temperature profile to see the consequences of Brinkman number $B_{m}$. An enhancement in Brinkman number $B_{m}$ causes a significant increase in the temperature profile because higher values of Brinkman number lessen the heat conduction, which creates an increment in the temperature profile. It can be viewed in Figure 11 that higher values of Ψ reduce the temperature profile. For the single-phase profile, the results are maximum; however, the suspension of particles tends to diminish the temperature profile. Similar behavior has been observed for all the cases of the artery.

**Figure 12:**
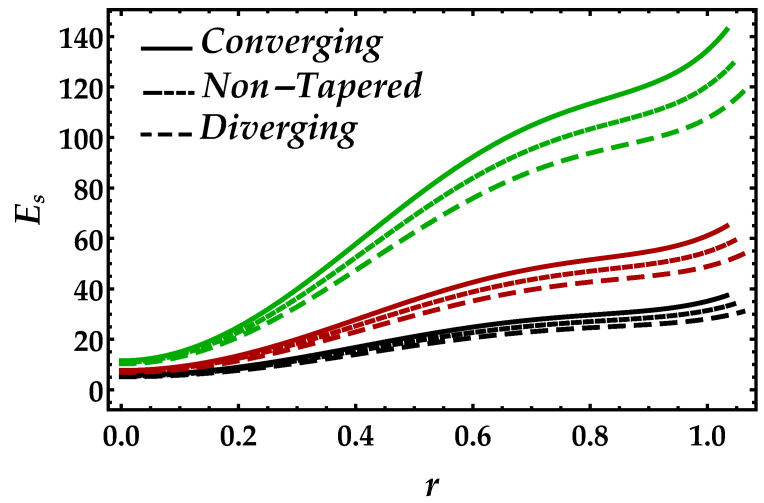
Entropy profile against multiple values of $B_{m}$. Black color: $B_{m}=0.15$; red color: $B_{m}=0.2$; green color: $B_{m}=0.3$.

**Figure 13:**
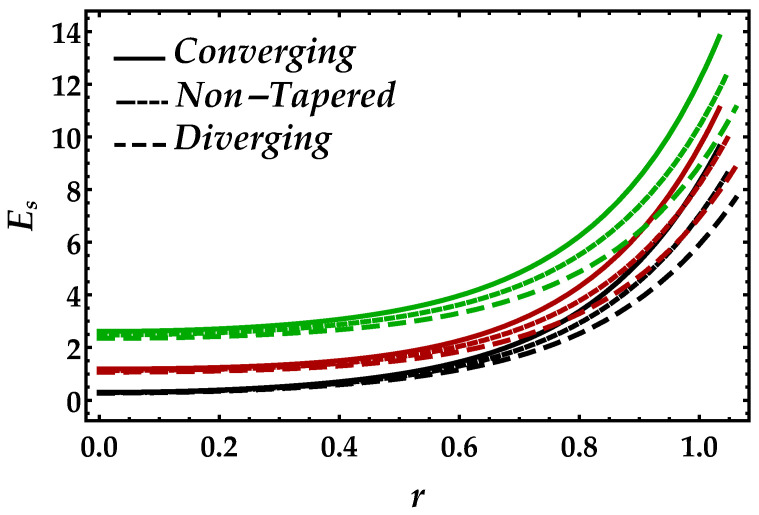
Entropy profile against multiple values of Ha. Black color: Ha=0.5; red color: Ha=1; green color: Ha=1.5.

Figure 12 and Figure 13 are developed to see the behavior of entropy $E_{s}$ against the Brinkman number $B_{m}$ and the magnetic parameter Ha. Figure 12 shows that, due to a significant increment, Brinkman number $B_{m}$ boosted the entropy profile, while for each case of the artery, it decreases. It depicts from Figure 13 that the presence of the magnetic also enhances the entropy profile, but the magnitude is small but remains uniform and positive throughout the domain.

**Figure 14:**
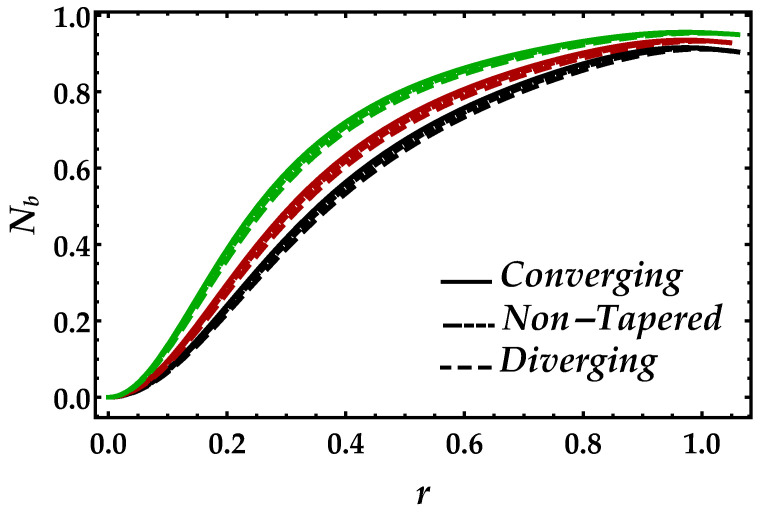
Consequences of Bejan number against multiple values of $B_{m}$. Black color: $B_{m}=0.15$; red color: $B_{m}=0.2$; green color: $B_{m}=0.3$.

**Figure 15:**
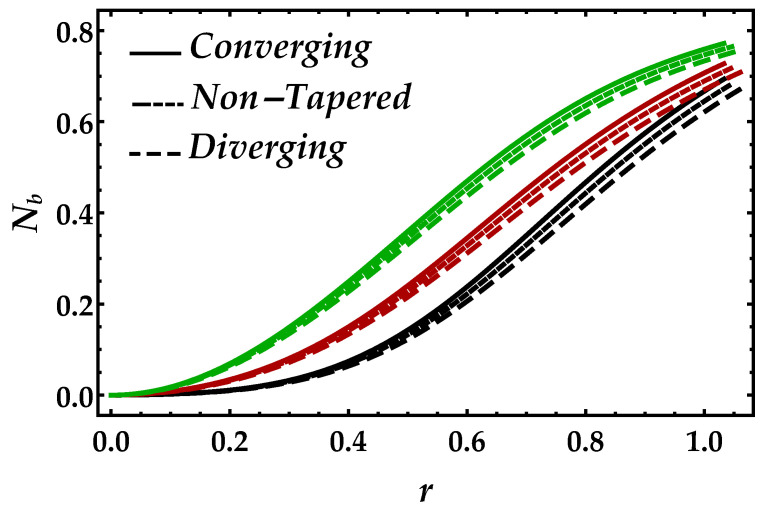
Consequences of Bejan number against multiple values of Ha. Black color: Ha=0.5; red color: Ha=1; green color: Ha=1.5.

Figure 14 and Figure 15 are sketched to examine the Bejan number $N_{b}$ profile against the Brinkman number $B_{m}$ and the magnetic parameter Ha. It can be seen in Figure 14 that Brinkman number $B_{m}$ convexly enhances the Bejan number. However, during the variation of Brinkman number, the converging, non-tapered and diverging arteries show themselves to be less effective. In Figure 15, the magnetic field enables the Bejan number to perform as an increasing function.

## 6. Conclusions

We have studied the entropy generation on the blood flow using the Jeffrey fluid model propagating through an anisotropically tapered artery under the suspension of magnetic Zinc-oxide (ZnO) nanoparticles (NPs). The proposed fluid model is incompressible and electrical conducting. Using the lubrication approach and the second law of thermodynamics, mathematical modeling is performed. The exact solutions are found using the computational software, *Mathematica*. The physical effects of all the leading parameters are discussed using the graphical method. The critical outcomes of the present analysis are summarized below:

(i) It is found that the magnetic field opposes the fluid motion in the middle of the artery while the nanoparticle volume fraction enhances the motion.
(ii) The magnitude of the Newtonian fluid velocity is lower compared with the non-Newtonian case.
(iii) The magnetic boosted the impedance profile, whereas the nanoparticle volume fraction opposes the impedance profile.
(iv) Temperature profile gets significantly increased due to the increment in Brinkman number and magnetic field.
(v) The enhancement in nanoparticle volume fraction reduces the temperature profile.
(vi) Entropy profile shows a uniform and increasing behavior against the magnetic field and Brinkman number.
(vii) Bejan number profile also rises due to the increment in the magnetic field and Brinkman number.
(viii) The monitoring of the blood temperature in the case of the tapered artery has supreme importance in controlling the temperature of blood in the living environment.
(ix) The presence of a magnetic field is advantageous to manage and control the blood motion at different temperatures.
(x) ZnO-NPs have promising applications in biomedical engineering due to its low toxicity, economically reliable, and excellent biocompatibility. ZnO-NPs also emerged in medicine i.e., the antibacterial and anticancer fields, and are also beneficial in antidiabetic treatment.

Limitations and future perspectives: There is no question that the usage of magnetic NPs is helpful and plays an essential role in the treatment of different diseases, including cancer. In particular, magnetic hyperthermia and magnetic drug delivery aggregate auspicious technologies for the treatment of cancer. However, the limitations are related to the strength of the extrinsic magnetic field and the problems associated with the penetration of the tissues, which have to have to be further enhanced. Moreover, the present results show the laminar flow description. The present study ignores the effects of shear-thinning and shear-thickening, which can be further elucidated in the near future. The proposed outcomes are hopefully beneficial for the experimental investigation of magnetized fluid flows with non-Newtonian models and heat transfer.
